# Immune response and potential therapeutic strategies for the SARS-CoV-2 associated with the COVID-19 pandemic

**DOI:** 10.7150/ijbs.66369

**Published:** 2022-02-14

**Authors:** Xianghui Li, Yabo Zhang, Libing He, Jiangzhe Si, Shuai Qiu, Yuhua He, Jiacun Wei, Zhili Wang, Longxiang Xie, Yanzhang Li, Tieshan Teng

**Affiliations:** 1 Institute of Biomedical Informatics, School of Basic Medical Sciences, Henan University, Kaifeng 475004, China.; 2 Henan International Joint Laboratory of Nuclear Protein Regulation, School of Basic Medical Science, Henan University, Kaifeng, Henan 475004, China.

**Keywords:** COVID-19, SARS-CoV-2, immunity, potential drugs

## Abstract

Following onset of the first recorded case of Coronavirus disease 2019 (COVID-19) in December 2019, more than 269 million cases and over 5.3 million deaths have been confirmed worldwide. COVID-19 is a highly infectious pneumonia, caused by a novel virus called severe acute respiratory syndrome coronavirus 2 (SARS-CoV-2). Currently, it poses a severe threat to human health across the globe, a trend that is likely to persist in the foreseeable future. This paper reviews SARS-CoV-2 immunity, the latest development of anti-SARS-CoV-2 drugs as well as exploring in detail, immune escape induced by SARS-CoV-2. We expect that the findings will provide a basis for COVID-19 prevention and treatment.

## Introduction

In December 2019, China reported a few cases of acute atypical respiratory disease in Wuhan, which rapidly spreads across the country. On February 11, 2020, World Health Organization (WHO) nominated the acute respiratory infectious disease “COVID-19” 1, while the International Committee on Taxonomy of Viruses (ICTV) immediately named this virus isolate “SARS-CoV-2”. Coronavirus, a spherical or polymorphic RNA virus, contains a single-stranded sense RNA genome and is the longest RNA virus so far reported 2. According to the systematic classification of viruses, coronavirus is part of Coronaviridae genus, the order Nidovirales, and can be further grouped into four genera, α, β, γ and δ based on their genome sequence homology 3. As a large family of viruses, coronavirus specifically is associated with common flu and severe infection. Previously, seven coronaviruses had been reported to cause human respiratory diseases 4, including NL63, human coronavirus 229E, HKU1, and OC43, which specifically infect the upper respiratory tract with mild symptoms 5. The other three coronaviruses include the Middle East respiratory syndrome (MERS-CoV), severe acute respiratory syndrome (SARS-CoV), and the recent SARS-CoV-2, which are capable of infecting the lower respiratory tract, as well as may bring about fatal pneumonia. SARS-CoV-2 represents different similarities to SARS-CoV (79%), bat coronavirus RaTG13 (96.2%), and pangolin coronavirus (92.4%) 6, 7. Herein, we explore immune reaction of SARS-CoV-2 infection together with the latest development of antiviral drugs in view of the recently published literature.

## Innate immune response to SARS-CoV-2 infections

SARS-CoV-2 invades the host cell where it undergoes massive proliferation. Consequently, the cell lyses, and then releases virus particles and intracellular components. The pattern recognition receptors (PRRs) in innate immune cells trigger the inducible response of innate immune system as soon as they recognize the virus particles and intracellular components 8. However, SARS-CoV-2 is also able to avoid or delay the stimulation of type I IFN-related response *in vivo*.

### Entry point of SARS-CoV-2

The spike protein of SARS-CoV-2 mediates major entry steps: including receptor binding and membrane fusion. Furin and TMPRSS2 are the two major proteases involved in viral attachment and membrane fusion 9. Furin, a ubiquitous proprotein convertase in the surface of Golgi apparatus, is capable of cleaving S protein into S1 and S2 subunits for virus maturation. Therefore, the S protein of the mature SARS-CoV-2 is comprised of two non-covalently subunits: the S1 subunit binds the ACE2 receptor and the S2 subunit anchors the S protein to the virion membrane and mediates membrane fusion. ACE2 engagement by SARS-CoV-2 will exposes an additional cleavage site in S2 subunits, termed S2' site. The S2' site can be cleaved by TMPRSS2 on the cell surface or cathepsin L in the endosomal compartment following ACE2-mediated endocytosis to release the fusion peptide and then initiate fusion pore formation, which allows the SARS-CoV-2 genome to reach the cytoplasm.

Although SARS-CoV-2 utilize ACE2 as the main receptor for cell entry, cells in the main target organs including the lungs and bronchi display low levels of ACE2 expression. Moreover, SARS-CoV-2 can also efficiently infect the upper respiratory tract and other tissues/organs, including the pharynx, heart, liver, brain, kidneys, and the gastrointestinal tract. These results suggest that SARS-CoV-2 employs other receptors for colonization and entry 10-12. Recent studies have demonstrated that a few cell surface molecules consisting of AXL, KREMEN1, CD147, KIM-1, TfR and DPP4 also are potential alternative receptor independent of ACE2. Meanwhile, heparan sulfate, GRP78, lectin and neuropilin-1 are considered as auxiliary attachment receptors dependent on ACE2 (Table 1).

### Recognition of SARS-CoV-2 infection

**Figure 1:**
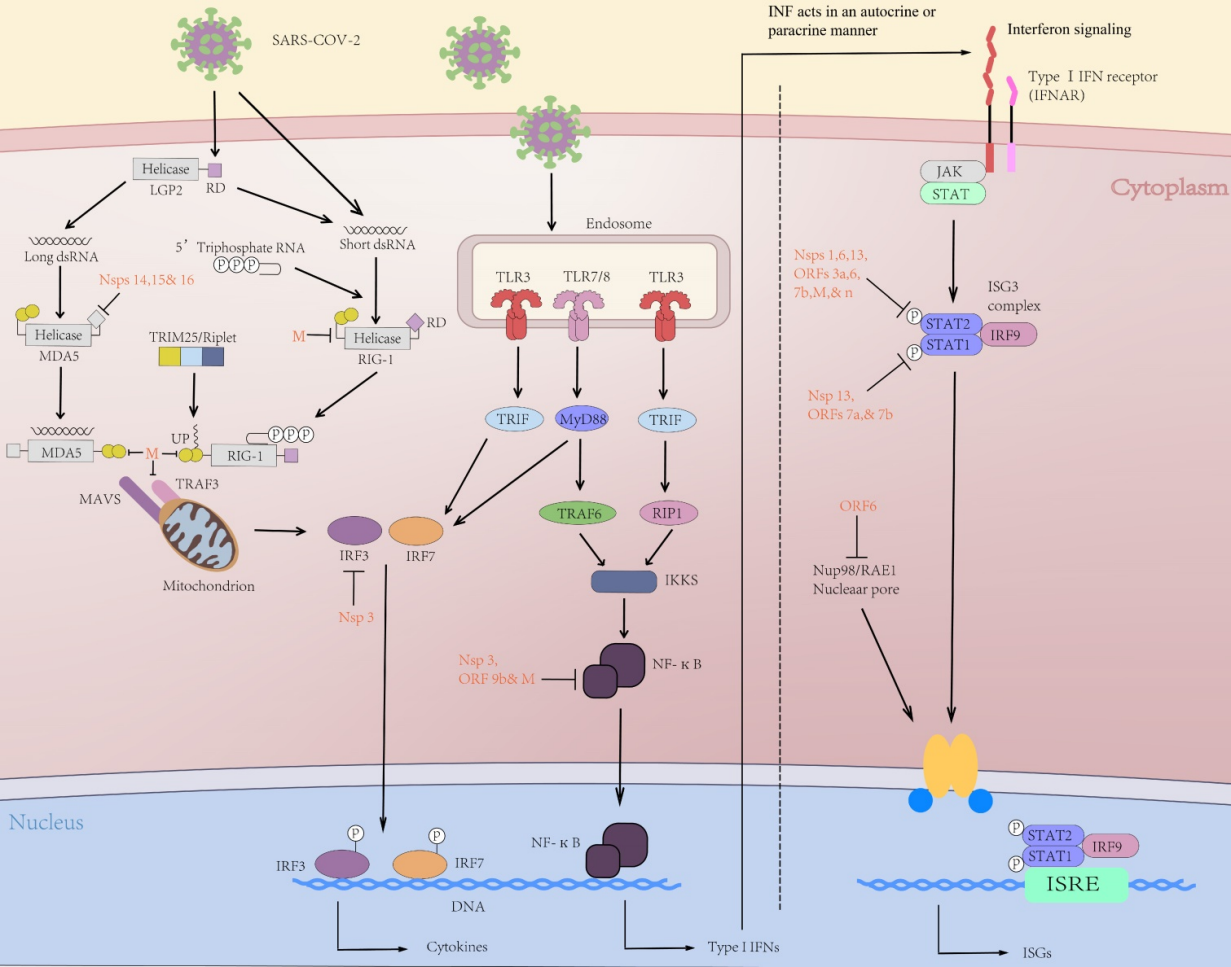
** Initial immune response to SARS-CoV-2 infection.** After the initial immune cell infection, RIG-I and MDA5 can trigger the activation of the adaptor molecule MAVS on the mitochondrial membrane, thus activating TRAF3 upstream of IFN regulatory factors (IRFs) 3/7. In the nucleus, NF-κB up-regulates expression of genes encoding interferon, pro-inflammatory cytokines (IL-1, IL-6, TNF-α, etc.) and chemokines that promote transcription of inflammatory acute phase-related proteins. The tyrosine kinases JAK1 and STAT were activated through the binding of Type I interferon and IFNAR. Activated JAK1/STAT can phosphorylate STAT1 and STAT2 to form heterodimers. ISGF3 is then transferred to the nucleus and initiate interferon-stimulated genes (ISGs) transcription. The ISG-encoded proteins exert antiviral activity by preventing virus invasion, replication, and budding. SARS-CoV-2-encoded proteins (red) inhibit multiple aspects of these pathways, resulting in decreased IFN and altered proinflammatory cytokine expression.

Following the human infected by SARS-CoV-2, virus genomic RNA or the intermediate product of virus replication (dsRNA) is recognized by the immune system via pattern recognition receptors (PRRs) 28. In PRRs, toll-like receptors (TLRs), RIG-I-like receptors (RLRs), for instance, melanoma differentiation-associated protein 5 (MDA5), and retinoic acid-induced gene protein I (RIG-I) are crucial in viral RNA recognition 29. Subsequently, the activated receptors cause a cascade of downstream immune signals, including IRF3/7 activation and nuclear translocation of NF-κB (nuclear factor kappa B), which thus can induce the expression of interferon (IFN), proinflammatory cytokines and chemokines, etc. (Figure 1) 30. The chemokines mentioned above are important mediators for giving rise to the release of innate immune cells from the bone marrow tissue.

### Cellular innate immune response to SARS-CoV-2

SARS-CoV-2 infection of the human lung triggers lung inflammatory innate immune response, which results in the recruitment of innate immune cells including monocytes, neutrophils, NK cells, DCs to the lungs. These innate immune cells are capable of releasing cytokines and prime adaptive T and B cell immune responses.

Phenotypic studies of infiltrating monocytes in the bronchoalveolar lavage (BAL) of COVID-19 patients have revealed that a surge of CD169^+^ monocytes and depletion of CD16^+^CD14^-^monocytes were observed early after symptom onset 31, 32. CD169^+^ monocytes have a strong association with proinflammatory cytokines, including IFN-γ, MCP-2, IL-18 and IL-6 32. At later stages, CD16^+^ monocytes were present in patients with severe disease, dominated by high CCL3 and CCL4 abundance 32. In the same way, the high concentration of neutrophil-released NETs (Neutrophil extracellular traps) are detected in COVID-19 patients' tracheal aspirate, which are considered to be related to the COVID-19 severity 33. SARS-CoV-2-triggered NETs characterized by extracellular exposure of DNA, myeloperoxidase (MPO) and histone, capacity to induce tissue damage and promote systemic immunothrombosis 33, 34.

It is widely reported that overall NK cell numbers are substantially reduced in the blood of COVID-19 patients relative to healthy controls 31. NK cells exert anti-viral activity but are functionally impaired with increased IFN-γ and TNF production in severe COVID-19 35. Further, NK cell dysfunction may be relevant to the development of fibrotic lung disease, as NK cells exhibited impaired anti-fibrotic activity 35.

As specialized producers of IFN-I, pDCs can secrete a great quantity of IFN-I isoforms immediately after SARS-CoV-2 infection. Notably, several days after SARS-CoV-2 infection, pDCs tune down their capacity for IFN-I production, which can favor prolonged viral replication and opportunistic secondary infections, termed “pDC exhaustion” 36. Furthermore, the expression of CD86 and HLA-DR in peripheral mDCs are reduced in severe COVID-19, which are crucial for antigen presentation and the induction of helper T cell response 37. This result may imply that the decreased DC capacity in response to TLR stimulation is associated to the diminished virus-specific T cell response in severe COVID-19 cases 37.

Cell transcriptomic profiling demonstrated that SARS-CoV-2 infection result in the activation of alveolar macrophages which promote the production of T cell chemoattractant 38. Then, T cells recruited by chemokines are capable of inducing the production of IFN-γ, which can in turn stimulate macrophages to produce a large number of inflammatory cytokines and further promote T cell activation 38. In each new area of infection, positive feedback loop between alveolar macrophages containing the virus and activated T cells could promote persistent injury and inflammation.

## Adaptive immune response induced by SARS-CoV-2

Adaptive immune response has been shown to play important roles in the control of almost all viral infections and promoting viral clearance, which consist of three major cell types: CD4^+^ T cells, CD8^+^ T cells and B cells. During SARS-CoV-2 infection, initiation of adaptive immune responses (6-10 days) was indispensable for naive cells undergoing proliferation and differentiation into effector cells 39. Meanwhile, the adaptive immune response will be not triggered by the time of the innate immune activation, which will result in high viral burden 40 and excessive lung immunopathology 41.

### CD4+ T cells

CD4^+^ T cell immune responses have close relationships with control of mainly SARS-CoV-2 infection more than CD8^+^ T cells 42-44. Among the COVID-19 cases in recovery period, almost all infections caused by SARS-CoV-2 proteins could initiate CD4^+^ T immune cell responses 42. And S, M, and N proteins were the most prominent targets of SARS-CoV-2-specific CD4^+^ T cells. Notably, compared with B and CD8^+^ T cells, the elevated levels of CD4^+^ T cells in acute COVID-19 had the strongest association with mild disease and rapid viral clearance 45. In contrast, rapid reduction of CD4^+^ T cells was related to severe COVID-19 46. T follicular helper cells (Tfh) and Th1 cells are mainly cell subtypes differentiated from CD4^+^ T cells. Th1 cells exert antiviral activities via IFN-γ production and secretion of cytokines IL-12, IL-3 and IL-2. Further, Th1 cells can induce CTL differentiation and activate B cells to secrete cytokines IL-10, IL-5 and IL-4. Tfh cells are able to assist differentiation of memory B cells and maintain long-lasting humoral immunity which is important for the progress of neutralizing antibody response 47.

### CD8+ T cells

CD8^+^ T cells have an important role in prevention of viral infections, on account of the availability of eliminating infected cells. In virus infections, the presence of virus-specific CD8^+^ T cells is closely related with the better outcome of patients with COVID-19 48. Several viral antigens consisting of S, N, M and Orf3a can be specifically recognized by SARS-CoV-2 specific CD8^+^ T cells 49, 50. In acute COVID-19, viral specific CD8^+^ T cell responses were activated swiftly, with a study of specific CD8^+^ T cells as early as the first day after COVID symptom onset 51. In acute SARS-CoV-2 infection, virus-specific CD8^+^ T cells indicate high expression of molecules related with potent cytotoxic effector functions consisting of CD107a, perforin, granzyme B and IFN-γ 51.

### Antibodies and B cells

Most SARS-CoV-2 infected persons seroconversion occurred were within 5-15 days of PSO, especially about 90% within 10 days of PSO 52-54. The main antigens involved in detection of seroconversion in infected individuals are Spike and nucleocapsid proteins 55. It is relatively easy to synthesis neutralizing antibodies against SARS-CoV-2 by many B cells with little affinity maturation required 56-59. There is a positive correlation between neutralizing-antibody titers and COVID-19 disease severity in large-scale cohort studies 60. Both SARS-CoV and MERS share the similar observations 61. After infection, the adaptive immune response occurs late, and many host cells are actually infected until an antibody response develops. This may be why the titer of SARS-CoV-2 neutralizing antibody is not associated with the reduction of disease severity in primary COVID-19 62.

## Dysfunctional immune responses to SARS-CoV-2

Severe COVID-19 is usually associated to a dysregulated innate immune response, which is widely characterized by a delayed IFN response relative to peak virus replication and an excessive inflammatory response. After SARS-CoV-2 invasion, timely activation of IFN-I is critical for suppression of viral replication 63. Notably, SARS-CoV-2 produces a variety of effector molecules, including structural proteins, non-structural proteins and accessory proteins (Table 1), which selectively counteract the IFN response, thereby evading the immune system 31. Although the specific molecular mechanisms remain to be determined, suppression of the IFN-I is considered to occur at several stages consisting of inhibiting the PRR recognition of SARS-CoV-2 RNA and preventing translation via degradation of host cell mRNA 64. The resultant suppressed or delayed IFN-I cascade enhances viral replication and causes inflammatory cytokine storm 65. Recent study found that cytokine profiles from COVID-19 patients with cytokine storm syndrome (CSS) were linked to lung damage, multi-organ failure, and a hostile prognosis in serious COVID- 19 patients 66, 67.

Based on the strong association between the increased cytokine levels and severity of COVID-19, modulation of inflammatory cytokines provides therapy strategies to mitigate severe disease. Cytokine-targeted therapies have become the preferable option as they have fewer potential adverse effects compared to conventional antiviral therapy, for instance JAK/STAT inhibitors, IL-1R inhibitors and IL-6R inhibitors 68, 69.

## Therapeutic drugs

**Figure 2:**
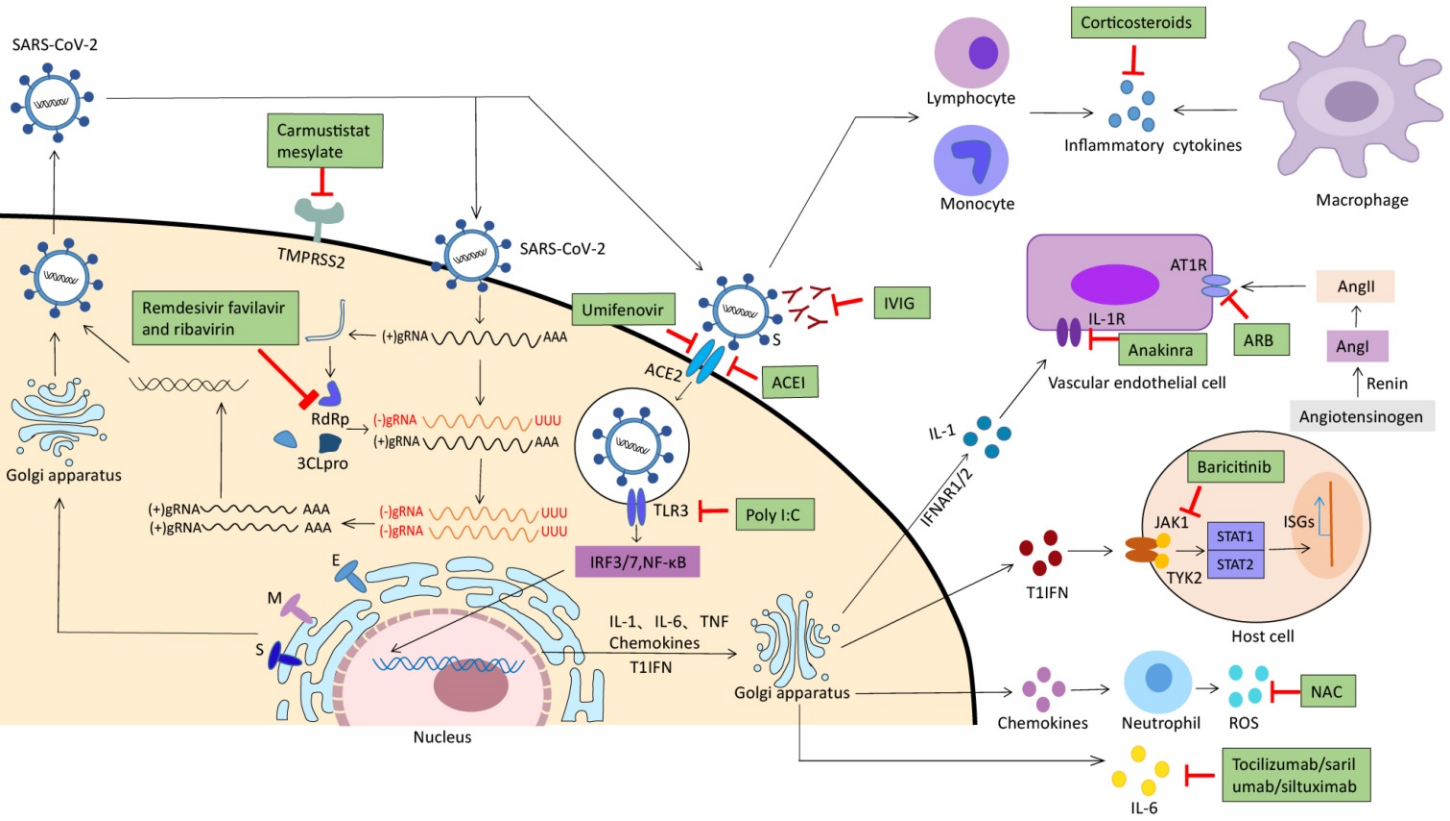
** Potential therapeutic strategies for virus receptor binding, replication proliferation and immune response.** Potential antivirals target the different steps of SARS- CoV-2 infection, ranging from receptor binding, entry and fusion to replication. Camostat mesylate, inhibits TMPRSS2; Remdesivir,favipiravir and ribavarin, inhibits RdRp; Umifenovir, inhibits S-ACE2 interaction and membrane fusion; Corticosteroids, inhibits inflammatory cytokines and neutrophil infiltration; Tocilizumab,sarilumab and siltuximab, binds IL-6 specifically and inhibits IL-6 signalling; IVIG, neutralizes the virus; Poly I:C, inhibits TLR3; NAC, scavenges ROS; ACEI, inhibits S-ACE2 interaction and membrane fusion; ARB, inhibits angiotensin II type 1 receptor(AT1R); Anakinra, inhibits IL-1 receptor.

No specific drug is available for COVID-19 cure based on the current understanding of the pathophysiology 86. The drug treatment options mainly focus on broad-spectrum antiviral drugs, membrane fusion inhibitors, protease inhibitors, virus spike protein targeted drugs, and immune modulators, etc. 87, 88. These drugs can prevent the virus interaction with the host cell receptor, or inhibit virus replication as well as excessive antiviral immune responses. Several potential anti-SARS-CoV-2 drugs are currently in clinical trials with different phases (Figure 2).

### RNA polymerase-RNA dependent inhibitors

Nucleoside analogues, such as remdesivir (GS-5734) 89, favipiravir 90 and ribavirin 91 are presently considered as potential drug candidates for the treatment of COVID-19. All these drugs are available for blocking viral RNA-dependent RNA polymerase (RdRp) during RNA synthesis, as a mechanism to block virus replication 92. Remdesivir, a broad-spectrum anti-virus' drug, was originally used for the treatment of Ebola and Marburg virus infections. And several animal studies have indicated that it is highly effective against the coronaviruses 93. A clinical study revealed that remdesivir exerts anti-virus activity against SARS-CoV-2 through *in vitro* and *in vivo* test 94, 95. Preliminary results of clinical trials showed that remdesivir could shorten the recovery time of COVID-19 patients by a couple of days compared to placebo, with no significant difference in mortality 96-98. FDA has authorized remdesivir for emergency use in severe COVID-19 cases 99. However, some international phase III clinical trials are currently evaluating the safety and efficacy of remdesivir for COVID-19 management. Favipiravir (T-705), an antiviral drug developed in Japan, has been approved in China, Russia, and India for COVID-19 treatment 100. A clinical study in China suggested that favipiravir significantly decreased the signs on chest imaging and shortened the time interval of virus infection 101. Moreover, a preliminary report from Japan showed that mild COVID-19 patients exhibited clinical improvement rates on the 7^th^ and 14^th^ day being 73.8% and 87.8%, respectively 102. Similarly, for severe COVID-19 patients, the clinical improvement rates on the 7^th^ and 14^th^ day were 40.1% and 60.3%, respectively 103. Also, there are several clinical trials on ribavirin for use in COVID-19 treatment. Although ribavirin has received extensive application in coronavirus treatment, it is associated with adverse reactions; therefore, it should be used with caution 104.

### Enzyme inhibitors prevent viral invasion

#### Camostat mesylate

Human transmembrane protease-serine 2 (TMPRSS2) is an essential host cell protease for SARS-CoV-2 spike protein activation 105. Camostat mesylate, an effective inhibitor of TMPRSS2, is currently under investigation for its effectiveness in COVID-19 patients. A possible advantage of blocking a critical host component like TMPRSS2 rather than targeting the virus itself is that its effect is not likely to be negated by mutations in the virus genome 106. Camostat mesylate is available for blocking SARS-CoV invasion and protects mice from lethal infection via inhibiting TMPRSS2 107. Recently, Clinical observation demonstrated that Camostat mesylate blocked SARS-CoV-2 entry into human lung cells 108. In addition, a few animal trials have exhibited that the treatment of SARS-CoV-2-infected mice with a therapeutic dose of Camostat mesylate can reduce the mortality of mice from 100% to 35% 109. Furthermore, these studies also revealed that Camostat mesylate reduces TGF-β levels which would alleviate COVID-19-induced acute respiratory distress syndrome (ARDS). Obviously, as a potential antiviral drug against SARS-CoV-2 infection, sufficient clinical data are still needed to support its efficacy.

#### Baricitinib

Baricitinib, often used to treat rheumatoid arthritis previously, was urgently approved by FDA to be used in combination with remdesivir in the treatment of patients with COVID-19 in November19, 2020. Baricitinib is available for inhibiting SARS-CoV-2 entry into target cells via preventing AP2-associated protein kinase 1(AAK1) and cyclin G-related kinase (GAK) activation 110-112. Moreover, Baricitinib is also available for reducing IL-6 levels and down-regulating CD80/CD86 expression in human monocyte, thereby impeding the release of type I IFN 113, 114. Collectively, Baricitinib can prevent cytokine storm syndrome via inhibiting the release of these cytokines, demonstrating that Baricitinib could be essential at every viral infection stage. Ultimately, the virus entry into cells in the early stage is reduced and then exerts anti-inflammatory effects in the late period 115.

#### ACEIs/ARBs

A large number of studies have shown that renin-angiotensin system (RAS) deregulation is responsible for ARDS, which is the main clinical manifestation of severe COVID-19. Although excessive angiotensin II is responsible for the exacerbation of COVID-19, yet the reduction of angiotensin II or blocking RAS pathway is able to limit the severity of acute lung injury caused by SARS-CoV-2 infection. Angiotensin-converting enzyme inhibitors (ACEIs) and angiotensin receptor blockers (ARBs) are considered to exert inhibition effects on the renin-angiotensin system (RAS) 116. Zhang *et al* found that the mortality of COVID-19 sufferers treated with ACEIs/ARBs was much lower than those treated without ACEIs/ARBs 117. Several clinical trials have also demonstrated that the up-regulation ACE2 expression levels induced by ARBs and ACEIs are supposed to reduce lung injury in SARS-CoV-2 infection presumably via decreasing ACE-derived Ang II 118, 119. While several organizations have issued statements advising the continuation of treatment of COVID-19 with ACEIs/ARBs, more large-scale clinical studies are still warranted to elucidate whether it could be associated with other unknown damages to the human body 120.

### Membrane fusion inhibitor

Umifenovir (arbidol), a derivative of indole carboxylic acids used for the treatment of influenza A and B virus infection, has been applied to in the treatment of SARS-CoV-2 infections 121. Umifenovir was available for inhibiting the interaction of S protein with ACE2 via incorporating into cell membranes and disturbing with the hydrogen bonding network of phospholipids, which blocks the membrane fusion of the virus. A study determined that Umifenovir monotherapy was superior to Liponavir/Ritonavir against COVID-19^122^. COVID-19 patients treated with Umifenovir and Liponavir/ritonavir exhibited better prognosis compared with patients treated with Liponavir/Ritonavir alone 123. Paradoxically, a clinical study in Wuhan revealed that Umfenovir did not increase the clearance rate of SARS-CoV-2 or accelerate the recovery of patients in any way 124. Thereby, the effect of Umfenovir in the treatment of SARS-CoV-2 needs further clinical study.

### IL-6 receptor-specific antibodies

Abnormally high levels of IL-6 are an indicator of poor outcome in COVID-19 patients with pneumonia and ARDS. Anti-IL-6 biological drugs, such as tocilizumab and sarilumab, have recently been adopted to treat COVID-19 patients with pneumonia 125, 126. Tocilizumab is a recombinant humanized anti-human IL-6 receptor monoclonal antibody which is capable of blocking the membrane-bound IL-6 receptor (mIL6R), thereby inhibiting signal transduction 127. COVID-19 patients treated with tocilizumab showed significant fever reduction within several days, and 75% patients were observed a reduced oxygen requirement 128. Another IL-6 receptor antagonist, sarilumab, may potentially prevent cytokine release syndrome (CRS) and pulmonary symptoms in severe COVID-19 patients 93. The efficacy and safety of sarilumab in the treatment of hospitalized patients with COVID-19 were evaluated in a randomized, double-blind study, which illustrated that sarilumab was effective in critical COVID-19 patients, but not in severe patients 129.

### IL-1 receptor antagonist

Anakinra is a recombinant non-glycosylated human IL-1 receptor antagonist 130, 131. It is initially approved for the treatment of auto-inflammatory disorders characterized by excess cytokines production which sharing clinical and molecular characteristics with COVID-19 hyper-inflammation 132, 133. Thus, anakinra was used to test the clinical efficacy of treatment in critical COVID-19 patients. Gilles Hayem *et al* found that anakinra treatment prominently decreased the mortality of severe COVID-19 patients 130. Another research has also demonstrated that the early administration of anakinra significantly diminished the requirement for invasive mechanical ventilation in COVID-19 ICU patients 93. Also, preliminary findings of Randomized Evaluation of COVID-19 Therapy (RECOVERY) trial exhibited that anakinra combined with corticosteroids in COVID-19 treatment is more efficacious than the single use 134.

### Reactive oxygen species (ROS) inhibitor

N-acetylcysteine (NAC) is an L-cysteine precursor which directly scavenges free radicals, especially reactive oxygen species (ROS) 135. *In vivo* and *in vitro*, NAC has been successfully adopted to manage numerous diseases, including chronic inflammation, acetaminophen poisoning and asthma 136. For COVID-19 infection, NAC was demonstrated to decrease inflammatory cytokines serum levels significantly. Furthermore, NAC can elevate the level of glutathione (GSH) in human T cells and prevent the T lymphocytes apoptosis induced by virus infection 132. In a randomized trial, ventilator associated pneumonia (VAP) patients related to COVID-19 treated with NAC had a shorter duration of hospital stay and a higher incidence of full restoration. NAC may alleviate insulin resistance by vitamin D deficiency, revealing NAC's potential benefit for diabetics with COVID-19 disease 137. The adverse effects are uncommon including hypersensitivity reactions, pyrexia and low blood pressure 138, 139.

### Polyclonal IgG antibody

Intravenous immunoglobulins (IVIGs) are polyclonal IgG antibodies from the plasma of SARS-CoV-2-infected blood donors 140. They potentially regulate systemic inflammation via capturing the activated complement factors, blocking the Fc γ-receptors and inhibiting lymphocyte differentiation and activation. Several studies showed that the transition from early to late course of COVID-19 is accompanied by the raised immunoglobulin levels, which indicating that IVIG therapy might be conducive to COVID-19 patients' rehabilitation 141, 142. In another clinical study, treatment with IVIG for COVID-19 pneumonia within 48 h reduced ventilator days and shortened ICU and hospital length of stay, eventually decreased 28-day mortality 143. An open randomized clinical trial indicated that adjuvant IVIG therapy was associated with a significantly shorter median time to real-time polymerase chain reaction negativity compared to standard of care (7 vs. 18 days) 144. Collectively, higher titer polyclonal immunoglobulins from the plasma of convalescent patients and neutralizing antibodies from lung lavage fluid of severe COVID-19 patients are potentially new treatment options in the management of COVID-19 145.

### Others

#### Interferon type I

Interferon type I can induce the expression of various interferon stimulating genes (ISGs) through the JAK-STAT pathway which interferes with every virus replication step 73. Previous studies have reported that IFN-I could be an appealing option for SARS-CoV treatment 146. *In vitro*, study of experimental data indicated that the sensitivity of SARS-CoV-2 to IFN-I is higher than SARS-CoV. Several studies exhibited that the joint application of nebulized IFN-α2b, corticosteroids and lopinavir/ritonavir decrease mortality rate to zero in COVID-19 patients. A study involving about 100 COVID-19 patients exhibited that IFN inhalation was very effective in treating SARS-CoV-2 infection, lowing the risk of developing severe disease by 79% 147. These observations support the option for the emergency application of IFN-I to treat the early COVID-19 pandemic 148.

#### Mesenchymal stem cell

Mesenchymal stem cells (MSCs) are pluripotent stem self-renewed cells, also known as adult stem cells. In regenerative medicine, they can improve impaired tissues and organs by differentiating into mature cells to release various cytokines 149, 150. Lately, Leng *et al* discovered that MSCs graft improved lung function in COVID-19 patients with pneumonia 151 via decreasing the number of inflammatory cells and elevating the level of lymphocytes 152. Intravenous transplantation of hUC-MSCs for the treatment of severe COVID-19 exhibited that the 28-day mortality rate in the hUC-MSC treatment group was 0, whereas 10.34% in the control group 153. Meanwhile, MSC-derived exosomes were tested clinically for treatment of COVID-19 patients and the results showed that 71% of the patients recovered 154, 155. In addition, MSC-derived exosomes also exhibited prominent amelioration in neutrophil as well as CD4^+^ and CD8^+^ lymphocyte numbers, with a decline in C-reactive protein (CRP) and Ferritin 153. Based on the existed evidence, MSC and its exosomes appear to be an appropriate option for treating patients with COVID-19 pneumonia 156, 157.

#### Corticosteroids (dexamethasone/methylprednisolone)

Following SARS-CoV-2 invasion, type II alveolar epithelial cells release inflammatory signals to recruit leukocytes, resulting in a "cytokine storm". Corticosteroids can decrease capillary permeability and reduce leukocyte migration to inflammatory sites 158. Moreover, it can inhibit neutrophil apoptosis and aggregation 159. Dexamethasone and methylprednisolone are synthetic corticosteroids 160, and both can inhibit the transcription activation of pro-inflammatory cytokines and cell adhesion genes with an anti-inflammatory effect. In moderate to severe COVID-19 patients, methylprednisolone treatment with a short course significantly reduced escalation of care from ward to ICU 161. Surprisingly, a preliminary report from the United Kingdom showed that dexamethasone treatment is only beneficial to the survival of patients with severe COVID-19 requiring respiratory support, but not mild COVID-19 patients who do not require respiratory support. In two other Brazilian studies, dexamethasone or methylprednisolone treatment conferred survival benefits to hospitalized patients with moderate to severe acute respiratory distress syndrome or over 60 years of age 162. However, early pulse dose therapy or long-term use of high-dose corticosteroids may be related to delayed virus clearance and high mortality 163. Therefore, it is imperative to use low doses of corticosteroids in accordance with the severity and clinical indications of COVID-19 patients.

### Antithrombotic therapy

Several evidences suggest that hospitalized COVID-19 patients often suffer from an acute infection-related coagulopathy and elevated risks of microvascular thrombosis 164. Strategies to prevent thrombosis are of critical importance for reducing the high mortality rate in COVID-19^165^. Several antithrombotic drugs have been proposed as potential therapies to prevent COVID-19-associated thrombosis, including heparin, and dipyridamole, both of which also possess pleiotropic anti-inflammatory or antiviral effects 164, 166.

Heparins, including unfractionated heparin (UFH) and low molecular weight heparin (LMWH), were mostly used for anticoagulant therapy related to SARS-CoV-2 infection 164, 167. Compared to COVID-19 patients treated with high-dose of LMWH, the mortality of hospitalized patients who did not received any anticoagulant treatment or received low-dose LMWH, was 6.2-fold and 2.0-fold increased, respectively 168. Also, there is general agreement that thromboprophylaxis should be administered to all hospitalized patients with COVID-19 infection with the use of standard dose of UFH or LMWH 169.

Dipyridamole (DIP), an antiplatelet agent, is often used for thromboprophylaxis combined with vitamin K antagonists following mechanical heart valve replacement 170. Previous study regarding coagulation function exhibited that the higher levels of D-dimer was presented in severe COVID-19 patients, becoming more significant with disease progression 171.In analysis of 12 cases of COVID-19 patients with prophylactic anti-coagulation therapy, it was found that DIP treatment was related to significantly diminished D-dimer levels in comparison to control groups 166. Half a month after initiation of DIP supplementation, 3 of the 6 severe COVID-19 patients and all 4 of the mild COVID-19 patients were discharged from the hospital 166.

## Conclusion

COVID-19 is the third most deadly human coronavirus disease. While its mortality rate is lower than SARS-CoV and the MERS, COVID-19 is highly infectious and poses threat to global health in this century. More than one year after the start of the pandemic, there seems to be cautious hope for its control and vaccination programs. However, there is still an urgent need for effective drug therapy to prevent severe diseases and limit the long-term complications of COVID-19. We believe that effective drug therapy may require a variety of methods, including early antiviral treatment to prevent virus replication, immunomodulatory treatment to deal with the late high inflammatory state and anticoagulation to prevent the sequelae of venous thromboembolism and micro-thrombosis. In this paper, the characteristics of innate and adaptive immunity induced by SARS-CoV-2 are reviewed, and the current treatment strategies are summarized in detail.

Importantly, with the continuous in-depth study, a lot of research reports have displayed that age, sex and heredity were also risk factors for severe COVID-19 39, 172. Firstly, there is little possibility for the older to develop a coordinated adaptive immune response to SARS-CoV-2 than the young people 44. T cell response against virus infection is determined by the repertoire of naive T cells, which will be declined substantially with age 173. Compared with 20-year-olds, 65-year-olds in the United States have a 90-fold higher risk of COVID-19 death, as well as 75-year-olds have a 200-fold higher risk of death. Secondly, men with severe COVID-19 have slightly higher risk than that in women due to sex differences in immune responses, although no obvious functional differences have been found in the adaptive immune response 174. However, in some studies, men infected with SARS-CoV-2 produced higher antibody titer 175. Interestingly, 10% of the severe COVID-19 patients were IFN-I auto-antibodies, while among them more than 90% were male 176. Therefore, severe gender differences in COVID-19 can be attributed to differences in innate immune response of IFN-I. Lastly, early genomic and transcriptome studies exhibited that a gene cluster on chromosome 3 was considered as a genetic susceptibility locus in patients with respiratory failure 177. The immune-related genes loci on chromosome 3 consist of CCR1, CCR2, CCR9, XCR1 and CXCR6. Among them, CCR1 is identified as a receptor of several chemokines (CCL3, 5, 7 and CCL23) 178. In addition, CCR1 deficient mice could be conducive to the protection of this receptor against excessive inflammation and reduce susceptibility to viruses and fungi 179.

## Figures and Tables

**Table 1:** Receptors, co-receptors, and cofactors involved in SARS-CoV-2 entry

No.	Molecule	Function	Virus type	Functional annotation	Refs
1	AXL	Receptor	SARS-CoV-2	A potential alternative receptor independent of ACE2	13
2	KREMEN1	Receptor	SARS-CoV-2	A potential alternative receptor independent of ACE2	14
3	CD147	Receptor	SARS-CoV-2, SARS-CoV	A potential alternative receptor independent of ACE2	15
4	KIM-1	Receptor	SARS-CoV-2, SARS-CoV, MERS-CoV	A potential alternative receptor independent of ACE2	16
5	TfR	Receptor	SARS-CoV-2	A potential alternative receptor independent of ACE2	17
6	DPP4	Receptor	SARS-CoV-2, MERS-CoV	A potential alternative receptor independent of ACE2	18
7	Heparan sulfate	Coreceptor	SARS-CoV-2, SARS-CoV, MERS-CoV	Auxiliary attachment receptor, dependent on ACE2	19
8	GRP78	Coreceptor	SARS-CoV-2, MERS-CoV	Auxiliary attachment receptor, dependent on ACE2	20
9	Lectin receptors	Coreceptor	SARS-CoV-2, SARS-CoV, MERS-CoV	Auxiliary attachment receptor, dependent on ACE2	21
10	Neuropilin 1	Coreceptor	SARS-CoV-2	Auxiliary attachment receptor, dependent on ACE2	22, 23
11	Furin	Cofactor	SARS-CoV-2, MERS-CoV	Proteolysis of S protein at the S1/S2 site	24, 25
12	Cathepsins	Cofactor	SARS-CoV-2, SARS-CoV, MERS-CoV	Proteolysis of S protein at the S1/S2 and S2′ sites	26
13	TMPRSS2	Cofactor	SARS-CoV-2, SARS-CoV, MERS-CoV	Proteolysis of S protein at the S1/S2 and S2′ sites	27

**Table 2:** Innate immune antagonism by SARS-CoV-2 proteins

Protein	Function in Virus Life Cycle	Function in Immune Escape	Refs
NSP1	mRNA degradation	Inhibiting IFN-β production	70, 71
NSP3	polyproteins cleavage	Antagonizing IRF3 and NF-κB signaling pathways	70, 72
NSP6	Forming membrane vesicles	Inhibiting STAT1 phosphorylation	72, 73
NSP12	RNA-dependent RNA polymerase	Inhibiting IFN-β production	70, 74
NSP13	RNA helicase	Abolishing the TBK-1 phosphorylation	70, 73, 75
Nsp14	3'-5' exonuclease	RNA capping to evade PRR detection	70, 75, 76
Nsp15	Endonuclease	Degrading RNA to evade PRR detection	75, 77
Nsp16	Methyltransferase	RNA capping to evade PRR detection	78, 79
ORF3a	Perforating host cell membranes	Inhibiting STAT1 phosphorylation	70, 73
ORF3b	Inhibiting IFN-I production	Blocking IRF3 nuclear translocation	80
ORF6	Blocking the transmission of signals	Blocking IRF3 nuclear translocation	70, 73, 75
ORF7a	Inducing suicide of infected cells	Inhibiting STAT2 phosphorylation	73
ORF7b	Inducing apoptosis	Inhibiting STAT2 phosphorylation	73
ORF8	-	Blocking IRF3 nuclear translocation	70, 81
ORF9b	-	Disrupting MAVS/TRAF3/TRAF6 signalosome	82
S	Mediating virus entry into host cells	Inhibiting IFNβ production	75
M	Participating virus assembly	inhibiting STAT1 phosphorylation	83, 84
N	Packaging the viral genome into a nucleocapsid	Inhibiting ISG production	85
